# An artesunate pharmacometric model to explain therapeutic responses in falciparum malaria

**DOI:** 10.1093/jac/dkad219

**Published:** 2023-07-20

**Authors:** Sompob Saralamba, Julie A Simpson, Noppon Choosri, Lisa White, Wirichada Pan-Ngum, Arjen M Dondorp, Nicholas J White

**Affiliations:** Mahidol Oxford Tropical Medicine Research Unit, Faculty of Tropical Medicine, Mahidol University, Bangkok, Thailand; Centre for Epidemiology and Biostatistics, Melbourne School of Population and Global Health, The University of Melbourne, Melbourne, Victoria, Australia; Center of Data Analytics and Knowledge Synthesis for Healthcare, Chiang Mai University, Chiang Mai, Thailand; Department of Biology, University of Oxford, Oxford, UK; Mahidol Oxford Tropical Medicine Research Unit, Faculty of Tropical Medicine, Mahidol University, Bangkok, Thailand; Department of Tropical Hygiene, Faculty of Tropical Medicine, Mahidol University, Bangkok, Thailand; Mahidol Oxford Tropical Medicine Research Unit, Faculty of Tropical Medicine, Mahidol University, Bangkok, Thailand; Centre for Tropical Medicine and Global Health, Nuffield Department of Medicine, University of Oxford, Oxford, UK; Mahidol Oxford Tropical Medicine Research Unit, Faculty of Tropical Medicine, Mahidol University, Bangkok, Thailand; Centre for Tropical Medicine and Global Health, Nuffield Department of Medicine, University of Oxford, Oxford, UK

## Abstract

**Background:**

The artemisinins are potent and widely used antimalarial drugs that are eliminated rapidly. A simple concentration–effect pharmacometric model does not explain why dosing more frequently than once daily fails to augment parasite clearance and improve therapeutic responses *in vivo*. Artemisinins can induce a temporary non-replicative or ‘dormant’ drug refractory state in *Plasmodium falciparum* malaria parasites which may explain recrudescences observed in clinical trials despite full drug susceptibility, but whether it explains the dosing–response relationship is uncertain.

**Objectives:**

To propose a revised model of antimalarial pharmacodynamics that incorporates reversible asexual parasite injury and temporary drug refractoriness in order to explain the failure of frequent dosing to augment therapeutic efficacy in falciparum malaria.

**Methods:**

The model was fitted using a Bayesian Markov Chain Monte Carlo approach with the parasite clearance data from 39 patients with uncomplicated falciparum malaria treated with artesunate from western Cambodia and 40 patients from northwestern Thailand reported previously.

**Results:**

The revised model captured the dynamics of parasite clearance data. Its predictions are consistent with observed therapeutic responses.

**Conclusions:**

A within-host pharmacometric model is proposed in which it is hypothesized that some malaria parasites enter a temporary drug refractory state after exposure to artemisinin antimalarials, which is followed by delayed parasite death or reactivation. The model fitted the observed sequential parasite density data from patients with acute *P. falciparum* malaria, and it supported reduced ring stage activity in artemisinin-resistant infections.

## Introduction

The artemisinin derivatives are the cornerstone of current antimalarial therapies.^1^ These well-tolerated and safe antimalarial drugs are highly effective in killing both circulating and sequestered malaria parasites, but they are eliminated very rapidly from the body. Both artesunate and artemether are converted rapidly *in vivo* to the common biologically active metabolite, dihydroartemisinin (DHA).^2^ The elimination half-life of DHA is usually reported as less than 1 h.^2^ Despite this, in most treatment regimens, the artemisinin derivatives are given only once daily. Simple pharmacometric models, in which parasite killing is a direct function of plasma concentration, predict that longer exposures to the artemisinins should increase the parasiticidal effect and augment therapeutic responses.^3–6^ This was a motivation behind the development of more slowly eliminated synthetic peroxide antimalarial drugs. More frequent dosing was proposed as a solution to the reduced parasite killing associated with artemisinin resistance.^6,7^ However, administering the rapidly eliminated artemisinins more than once daily neither accelerates parasite clearance nor improves therapeutic responses.^8,9^ It has also been suggested that parasite clearance lags behind killing of malaria parasites, so damaged or dead parasites accumulate in the circulation relative to live parasites.^5,10^ From this hypothesis it was deduced that slowing of the widely measured parasite clearance rate^11^ may not be a suitable indicator of artemisinin resistance, and that the failure of split dosing to accelerate parasite clearance could obscure a benefit in cure rates.^5^ However, a meta-analysis of artemisinin and artesunate monotherapy trials showed that split dosing was not associated with higher cure rates.^9^ Thus, the results of clinical trials do not support the hypothesized dissociation between parasite killing and clearance. Taken together, the observed therapeutic responses following antimalarial treatment with artemisinins are not explained adequately by simple direct concentration-dependent malaria parasite killing. As a result, the mathematical models based on this simple pharmacometric relationship do not explain satisfactorily the malaria parasite density dynamics observed *in vivo* following treatment with artemisinin derivatives.^8,9^

More complex pharmacometric relationships must exist. Potentially reversible parasite injury and delayed parasite stress responses after drug exposure might both contribute to these more complex pharmacodynamics.^12–20^ Temporary drug insensitivity in growth-arrested malaria parasites is well recognized. The persistence of temporarily drug insensitive or ‘dormant’ parasites explains the failure of artemisinin monotherapies to achieve 100% cure rates, even with 7 day courses in artemisinin-sensitive infections.^12,13^

To accommodate these therapeutic observations we hypothesize that following exposure to artemisinin drugs, parasites can be damaged and rendered temporarily refractory to further injury, and that a fraction of these injured parasites could recover afterwards. We assessed whether incorporating this hypothesis into a mathematical model could satisfactorily describe parasite clearance dynamics after treatment with artemisinin derivatives.

## Methods

### Parasite clearance and pharmacokinetic data

We re-evaluated the data used to generate an earlier simpler mathematical model in which there was direct concentration-dependent malaria parasite killing.^4^ The data used in this evaluation were serial parasite count data (asexual parasite densities) and plasma drug concentration data (DHA) from published clinical studies conducted to characterize artemisinin resistance in falciparum malaria^21^ (ClinicalTrials.gov number NCT00493363, https://clinicaltrials.gov/ct2/show/NCT00493363; and ISRCTN number ISRCTN64835265, https://doi.org/10.1186/ISRCTN64835265). DHA is the main biologically active metabolite of artesunate and it is the principle contributor to the antimalarial effect.^2^ In these trials, informed consent was obtained from all subjects or their legal guardians. The study protocols of the clinical trials were reviewed and approved by the Ministry of Health in Cambodia, the Ethics Committee of the Faculty of Tropical Medicine of Mahidol University in Thailand, the Oxford Tropical Medicine Ethical Committee, and the Technical Review Group of the WHO Western Pacific Regional Office. All methods were performed in accordance with the relevant guidelines and regulations. The data were from 20 patients with acute falciparum malaria studied in Pailin, western Cambodia, and 19 patients studied in Wang Pha, western Thailand. These patients were treated with artesunate monotherapy as part of a clinical trial conducted during 2007 and 2008 and reported previously.^21^ Patients received 2 mg/kg oral artesunate every 24 h for 7 days. Parasite counts were determined by microscopy at 0, 4, 8 and 12 h, and then every 6 h until two consecutive negative slides were recorded. Plasma DHA concentrations were measured at 0, 0.25, 0.5, 1, 1.5, 2, 3, 4, 5, 6, 8 and 12 h following the first dose.^22^

### Mathematical model

The base model without antimalarial treatment is that of White *et al*. describing the within-patient dynamics of *Plasmodium falciparum* parasites.^23,24^ The age distribution of the parasites at any time before treatment was assumed to be unimodal and Gaussian. The asexual life cycle length was assumed to be 48 h. The age distribution of the infection shifts to the right as parasites become older. The infection multiplication factor is the population average number of merozoites that successfully infect new RBCs from one infected RBC. During the expansion phase of the infection this is a positive number, with an upper limit set by the average number of merozoites per schizont. During the expansion phase of the infection multiplication efficiencies in non-immune subjects are typically in the 20–50% range. This falls abruptly at high parasite densities. Time in the model was discretized and fixed at 1 h intervals. Asexual *P. falciparum* parasites were therefore divided into 48 individual age groups, from 1 to 48 h. Each hour the parasites move to the next hour age group until the final hour of the asexual cycle, 47–48 h. Then, following schizont rupture and merozoite invasion, the asexual cycle restarts with ring stage parasites aged 0–1 h at a new parasite density defined by the preceding density and the average multiplication factor.

### Antimalarial pharmacodynamics

Antimalarial drugs kill malaria parasites, but the pharmacodynamic effect depends both on the plasma concentrations of drug (exposure) and the susceptibility of the predominant stage of parasite development. During antimalarial drug exposure a fraction of asexual parasites at each susceptible stage of development will be damaged. Some of these die (and the damaged ring stages are subsequently ‘pitted’ from the infected RBC by the spleen).^11^ It is hypothesized that a fraction of the remainder can recover, but their development is temporarily arrested. During this arrest period the damaged parasites are refractory to further injury by the antimalarial drug. In the presence of the artemisinin derivative, the fraction of the damaged parasites, *f*(*t*), at time *t* is modelled by the Hill function of the plasma concentration of DHA, *c*(*t*):


(1) $$f(t)=\;\frac{E_{m}c{\left(t\right)}^{\gamma }}{c{\left(t\right)}^{\gamma }+EC_{50}^{\gamma }}$$

Where *E_m_* is the maximum extent (%) that the exposed parasites can be damaged by the drug, *c*(*t*) is the concentration of DHA at time *t* (h), *EC*_50_ is the concentration that gives 50% of the damage effect, and *γ* is the slope constant. Following oral artesunate administration, the parent drug is rapidly hydrolysed both in the gut and the plasma so only plasma DHA concentrations were modelled. Damaged malaria parasites that recover after a period of refractoriness return to their pre-treatment stage, with subsequent normal ageing, multiplication and pre-treatment drug susceptibility. Parasites die at a rate of *ν*(/h). Surviving parasites will mature to produce new merozoites. The fraction of damaged parasites that recover to their pre-treatment normal state at time *t*, *κ*(*t*), is calculated from


(2) $$\kappa (t)=\;\frac{r_{m}}{1+\exp\;(-f(t)\times (t-lag))}$$

**Figure 1.**
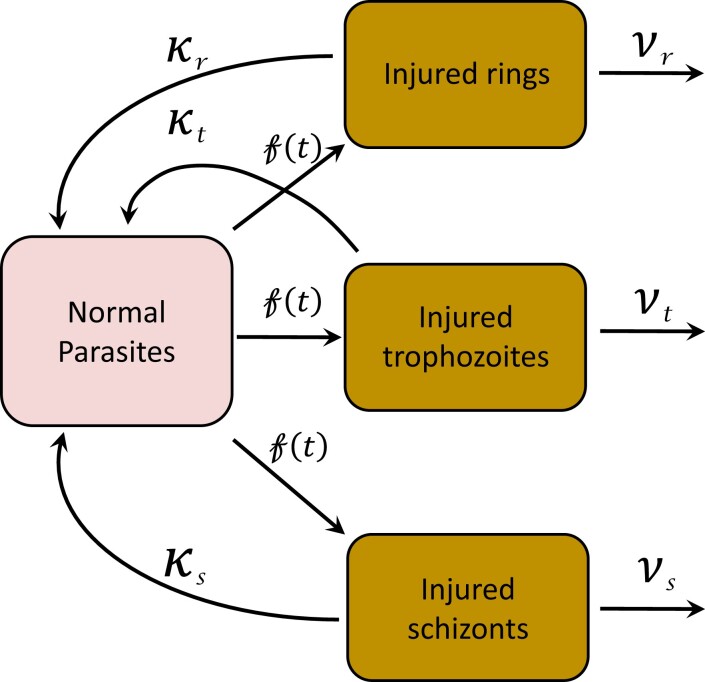
The antimalarial effect of artemisinins is proposed to operate in two sequential stages; first there is injury, during which the parasites are refractory to further drug-induced damage, followed either by parasite death and clearance or, in a small proportion, by recovery. The proposed model is based on the structure by White *et al*.^23^ and Saralamba *et al*.^4^ for the parasite ageing and age-specific interactions between artesunate (DHA) and *P. falciparum* parasites, respectively. The diagram shows the proposed model, where *f*(*t*) is the fraction of sensitive parasites at time *t* that can be damaged or injured, *κ* is the recovery rate of each asexual stage, and *ν* is the death rate of each asexual stage. This figure appears in colour in the online version of *JAC* and in black and white in the print version of *JAC*.

Where *r_m_* is the maximum fraction of the damaged parasites that can recover, *f*(*t*) is the fraction of damaged parasites at time *t*, and *lag* is the recovery lag time (the interval after the period of injury before the parasites resume their normal development). These recovery and death fractions are assumed to depend on the parasites’ stage of development. The susceptibility of malaria parasites to all antimalarial drugs is strongly dependent on their stage of asexual development. For simplicity we divide the asexual parasites into three developmental stages: rings (0–26 h); trophozoites (27–38 h); and schizonts (39–48 h). Laboratory studies suggest that young ring stages are most likely to enter a state of dormancy, particularly following exposure to artemisinin derivatives.^13–15^ A diagram of the model is shown in Figure 1. The plasma concentration of DHA, *c*(*t*), following each oral dose was modelled as follows:


(3) $$c(t)=\{\begin{array}{c} \left(\frac{c_{m}t}{t_{m}}\right),\;\;t\leq t_{m}\\ c_{m}e^{-k\;t},\;\;t>t_{m} \end{array},$$

Where *t_m_* is the time of maximum plasma concentration, *c_m_* is the maximum concentration, and *k* is the drug first-order elimination rate constant. DHA elimination kinetics are well characterized by a single rate constant.^2,22,25^

The pharmacodynamic model output was the number of circulating parasites calculated from the number of parasites at each age of development multiplied by the observable probability function of the parasites at different ages, as previously proposed by Saralamba *et al*.^4^ The Stan code of the model is available in the Supplementary information, available at *JAC* Online.

### Statistical analysis

The plasma concentration model in Equation (3) was fitted to the DHA plasma concentration profile of each patient separately following their first oral dose of artesunate (2 mg/kg). Model fitting was done in Wolfram Mathematica.^26^ The estimated concentration parameters (*t_m_*, *c_m_*, *k*) were used as inputs for generating the DHA plasma concentration at any time *t* following each dose during treatment for each individual patient.

The proposed pharmacodynamic model was fitted to the observed parasite count data obtained during artesunate monotherapy using a Bayesian hierarchical model approach for estimating the model parameters at both individual and population levels. The model parameters were transformed and reparametrized using the method proposed by Lesaffre *et al*.^27^ In this, the pharmacodynamic model parameters (*θ_ind_*) for each individual and their lower (*b_L_*) and upper (*b_U_*) bounds were transformed to the unbounded parameters $\varphi_{ind}=\ln\;\left(\frac{\theta_{ind}-b_{L}}{b_{U}-\theta_{ind}}\right)$. The unbounded population means were transformed from $\varphi_{\;pop}=\ln\;\left(\frac{\theta_{\;pop}-b_{L}}{b_{U}-\theta_{\;pop}}\right)$, where *θ_pop_* is the population mean of *θ_ind_*. In the sampling process, these individual parameters *φ_ind_* were reparametrized to be $\varphi_{ind}=\varphi_{\;pop}+\omega_{\;pop}L\eta$, where *ω_pop_* is the standard deviation, *L* is the lower Cholesky factor and η∼N(0,1).

The fitting was implemented in Stan using the Hamiltonian Monte Carlo (HMC) method. The likelihood function was derived assuming that the log_10_-transformed value of each observed parasite density measurement (Φ_i_) at time *i* was sampled from a normal distribution, of which the mean was the model output (M*_i_*) for that observed time *i* and its standard deviation was ϱ. That is, the likelihood can be written as:


(4) $$\log_{10}\;\Phi_{i}∼Normal(M_{i},\varrho )$$

The first 5000 parameter values sampled for each chain were discarded as burn-in and the subsequent 5000 samples from each chain (*n* = 3) were used to estimate the posterior distributions and also the posterior predictive checks. The convergence of the chains of each parameter was assessed using trace plots and the *Ȓ* statistic.^28^ The prior distribution of each model parameter was the uniform distribution with boundaries as shown in Table 1. The Stan code of the model is available at https://github.com/slphyx/DamagedParasites.

**Table 1.** Parameter definitions for the DHA pharmacodynamic model and the prior distributions chosen

Parameter description	Symbol	Prior distribution	Unit
Initial number of parasites in the body on admission (log_10_ scale)	*N*_0_	U(8–13)	parasites
Mean age of parasites on admission	*μ*	U(1–48)	h
Standard deviation of the age of parasites on admission	*σ*	U(1–48)	h
Parasite multiplication factor per asexual cycle	*pmf*	U(1–30)	parasites/48 h
Maximum extent (%) that the parasites can be injured by the drug	*E_m_*	U(50–99.99)	percentage/h
Plasma concentration of DHA that gives 50% of the injury effect	*EC*_50_	U(5–100)	ng/mL
Slope of the parasite injury response curve	*γ*	U(1.5–9.5)	
Recovery rate of each asexual stage^a^	*κ*	U(0–0.001)	/h
Death rate of each asexual stage^a^	*ν*	U(0–1)	/h
Lag time	*lag*	U(0–48)	h

The intravascular malaria parasite population is divided into three developmental stages: rings (0–26 h); trophozoites (27−38 h); and schizonts (39–48 h).

### Split-dose simulation

To predict the effect of administering artesunate every 12 h for 7 days to patients with falciparum malaria, the models were re-run using parameter values sampled from the posterior distributions estimated from fitting the model to parasite clearance profiles following artesunate monotherapy once daily, except that plasma drug concentration profiles were changed from once to twice daily drug administration. For each hypothetical patient, parasite clearance times were calculated from the parasite versus time profiles, which were sampled from the fitted hourly profiles of each patient. Here the parasite clearance time was defined as the time from first dose of artesunate to the time that the simulated total circulating parasite numbers were below the detection limit (L), which differed between the hypothetical patients and was estimated from:


(5) L≈l×80,000×weight

Where *l* is the lowest observed parasite density (parasites/µL), and weight is the recorded body weight of the patient (kg). All patients were assumed to have a total blood volume of 80 mL/kg.

To test whether the model could reproduce the trial results from Das *et al*.,^8^ which showed no difference in parasite clearance time whether patients were given 2 mg/kg artesunate every 12 or 24 h for 7 days, clearance times predicted by simulating both dose regimens were compared visually.

## Results

The measured plasma DHA concentration profiles^21^ of patients given 2 mg/kg oral artesunate daily were first analysed to estimate individual specific pharmacokinetic parameters. Tables S1 and S2 summarize the pharmacokinetic parameters estimated for DHA for the 39 patients. A sequential pharmacokinetic/pharmacodynamic modelling approach was then performed in which plasma DHA concentrations were simulated for each individual patient at the times corresponding to the parasite density measurements.

### Pharmacodynamic model

**Figure 2.**
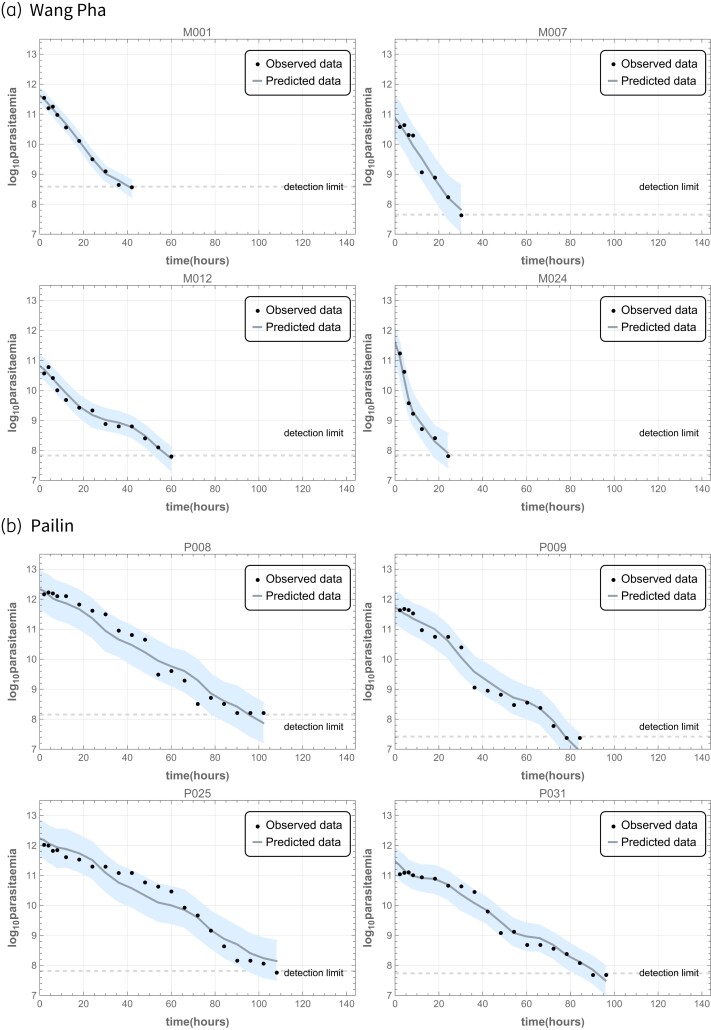
Examples of fitting the new pharmacodynamic model to the serial parasite count data from patients who received artesunate monotherapy in (a) Wang Pha, Thailand, and (b) Pailin, Cambodia. In each plot, the dots represent the observed data and the dark grey line represents the median of the model outputs. The light-grey shaded area represents the 95% credible intervals. See Supplementary information for plots of parasitaemia profiles for all 39 patients. This figure appears in colour in the online version of *JAC* and in black and white in the print version of *JAC*.

The proposed pharmacodynamic model was fitted to the parasite clearance profiles from patients treated with artesunate monotherapy, using a Bayesian hierarchical model.^29^ The posterior predictions showed that the model could reproduce the observed parasite dynamics during artesunate treatment (Figure 2 and Figures S1 and S3) and the Markov chains of each model parameter converged within the given iterations (*Ȓ* = 1). The estimated values of the population mean pharmacodynamic parameters are presented in Table 2.

**Table 2.** Posterior summaries for the population mean pharmacodynamic parameters calculated from 1000 draws from the posterior distribution

	Pailin	Wang Pha
Parameter	Posterior median (95% credible interval)	Posterior median (95% credible interval)
Initial number of parasites in the body on admission (log_10_ scale)	12.13 (11.76–12.49)	11.64 (11.29–11.94)
Mean age of parasites on admission (h)	18.98 (15.77–22.24)	16.23 (14.06–18.31)
Standard deviation of the age of parasites on admission (h)	8.43 (7.36–10.14)	5.82 (4.73–6.62)
Parasite multiplication factor (/48 h)	3.54 (1.042–19.93)	6.27 (1.26–23.10)
Slope of the injury response curve	6.11 (1.56–9.89)	6.52 (1.99–9.75)
Plasma concentration of DHA that gives 50% of the injury effect (ng/mL)	43.11 (5.30–85.46)	54.26 (6.18–95.65)
Death rate of rings (/h)	0.0267(0.0024–0.0822)	0.2890 (0.1188–0.4018)
Death rate of trophozoites (/h)	0.3429 (0.2250–0.4616)	0.3811 (0.2364–0.4649)
Death rate of schizonts (/h)	0.2187 (0.0283–0.4494)	0.3866 (0.1070–0.4963)
Recovery rate of rings (/h)	$4.786\times 10^{-5}$ $\left(2.779\times 10^{-7}\text{–}9.629\times 10^{-5}\right)$	$5.499\times 10^{-5}$ $\left(3.261\times 10^{-6}\text{–}9.482\times 10^{-5}\right)$
Recovery rate of trophozoites (/h)	$4.816\times 10^{-5}$ $\left(1.793\times 10^{-6}\text{–}9.593\times 10^{-5}\right)$	$5.543\times 10^{-5}$ $\left(1.642\times 10^{-6}\text{–}9.667\times 10^{-5}\right)$
Recovery rate of schizonts (/h)	$4.983\times 10^{-5}$ $\left(3.893\times 10^{-6}\text{–}9.797\times 10^{-5}\right)$	$5.696\times 10^{-5}$ $\left(3.186\times 10^{-6}\text{–}9.629\times 10^{-5}\right)$
Lag time (h)	2.01 (0.04–7.32)	2.42 (0.04–7.01)

**Figure 3.**
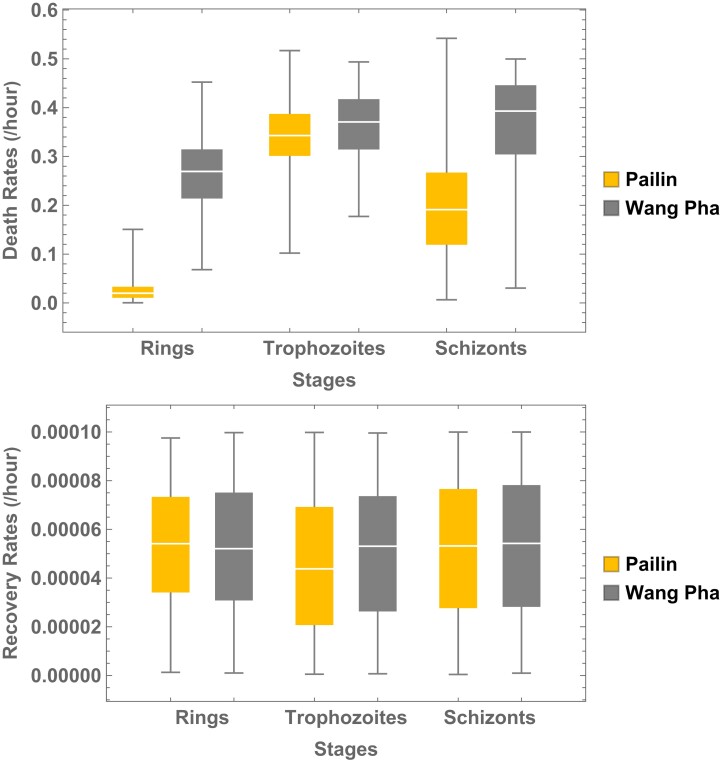
Box-and-whisker plots generated from the posterior distributions of estimated population means of the asexual stage-specific parasite death rates (top) and parasite recovery rates (bottom) for each study site (left = Pailin, western Cambodia; right = Wang Pha, western Thailand). This figure appears in colour in the online version of *JAC* and in black and white in the print version of *JAC*.

The clinical study^21^ was conducted in two areas, one where artemisinin-resistant *P. falciparum* parasites were prevalent (Pailin, western Cambodia) and the other where parasites were still sensitive to artemisinin (Wang Pha, western border of Thailand). The population means of estimated death and recovery rates of each parasite asexual stage indicated, as expected from current understanding of artemisinin resistance and evidence from *in vitro* studies, that the estimated death rates of ring stage parasites in Pailin were substantially lower (over 10-fold) than those in Wang Pha (Table 2). Estimated schizont death rates were about 45% lower in Pailin. In contrast, the estimated death rates for trophozoite parasites were not different between the locations. Parasite recovery rates following injury were slightly different between stages and sites. Recovery rates from Pailin, western Cambodia were slightly lower than those from Wang Pha, western Thailand. The box-and-whisker plots of recovery and death rates for ring, trophozoite and schizont stage *P. falciparum* parasites compared between the clinical sites are shown in Figure 3. The population means of the post-injury recovery lag times for damaged parasites to recover to their normal state were 2.42 (95% credible interval: 0.04–7.00) h in Wang Pha and 2.01 (95% credible interval: 0.04–7.32) h in Pailin.

### Split-dose simulation

**Figure 4.**
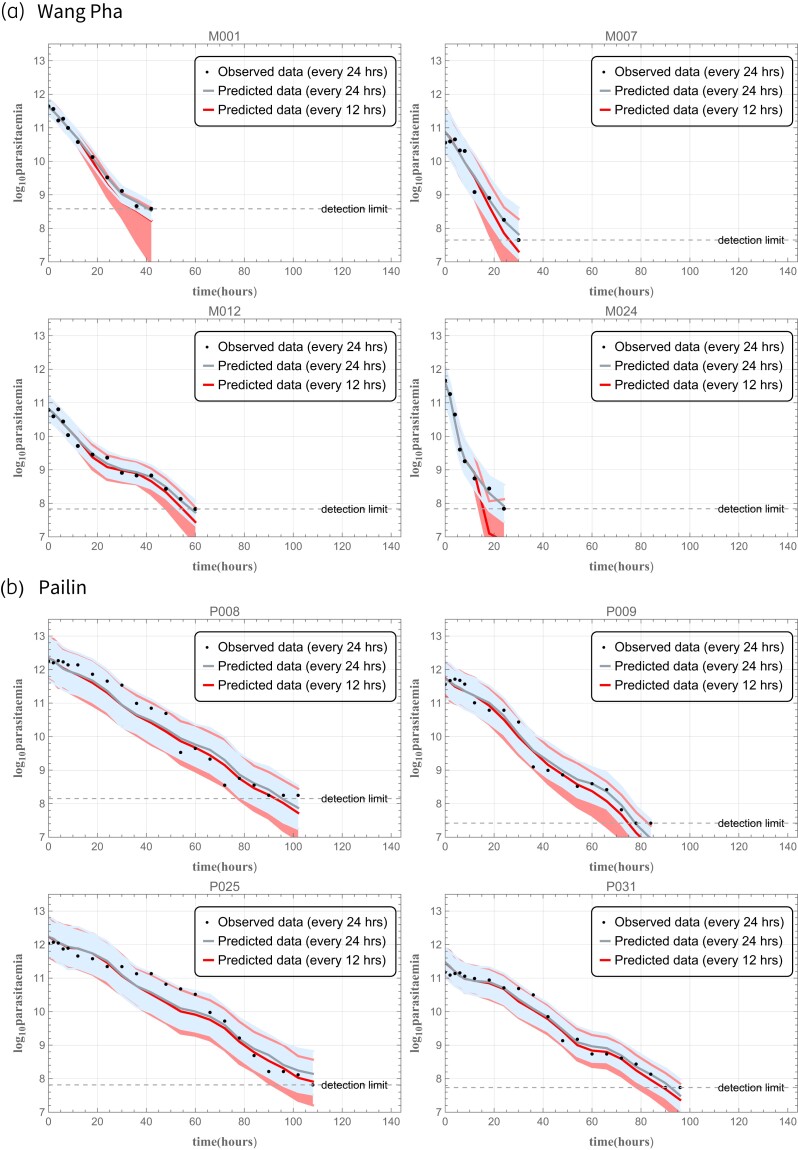
Examples comparing simulations of once-daily versus twice-daily artesunate administration in (a) Wang Pha, Thailand, and (b) Pailin, Cambodia. The dots represent the observed data, the purple lines represent the median of the model outputs for once-daily administration and the green lines represent the outputs for twice-daily administration. See the Figures S1–S4 for plots of parasitaemia profiles for all 39 patients. This figure appears in colour in the online version of *JAC* and in black and white in the print version of *JAC*.

**Figure 5.**
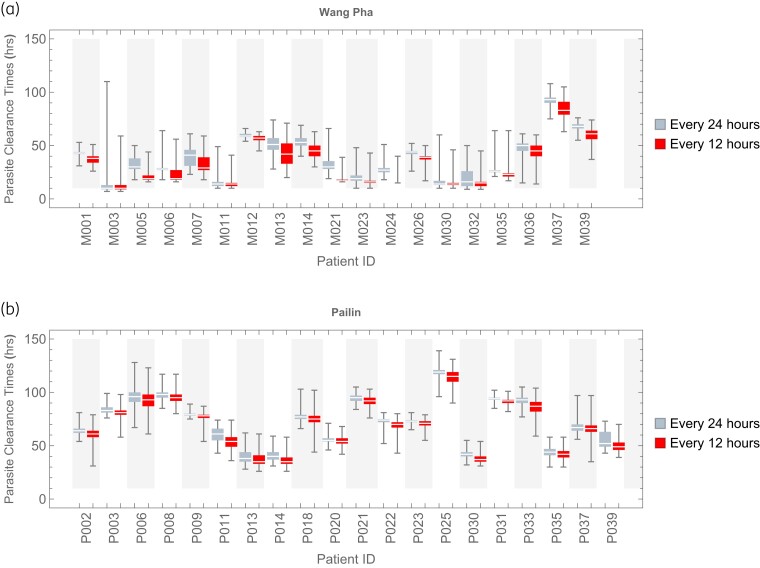
Comparison of parasite clearance times (h) derived from simulated parasitaemia profiles of hypothetical patients with acute falciparum malaria receiving 2 mg/kg artesunate either every 24 h (left) or every 12 h (right) for 7 days from (a) Wang Pha, western Thailand, and (b) Pailin, western Cambodia. This figure appears in colour in the online version of *JAC* and in black and white in the print version of *JAC*.

**Figure 6.**
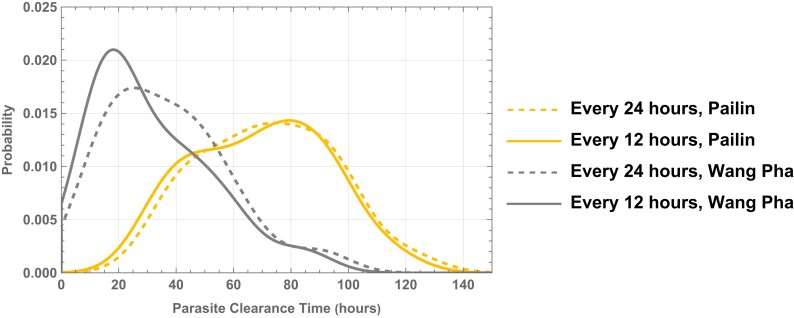
Comparison of distributions of parasite clearance times (h) derived from simulated parasitaemia profiles for once-daily (thick lines) versus twice-daily (dashed lines) administration for 7 days from Pailin, western Cambodia (right) and Wang Pha, western Thailand (left). This figure appears in colour in the online version of *JAC* and in black and white in the print version of *JAC*.

For the split-dose simulation, the DHA concentration profiles following individual doses were incorporated in the model every 12 h for 7 days (i.e. an oral artesunate dosing regimen of 2 mg/kg every 12 h for 7 days) and parasite clearance was estimated from the corresponding simulated parasitaemia profiles (Figure 4). Figure 5 compares the parasite clearance profiles of the daily and twice-daily artesunate dosing regimens. These distributions of parasite clearance show that, within the same clinical site, predicted parasite clearance profiles following once-daily and twice-daily drug administration are not significantly different (Figure 6 and Table S3).

## Discussion

Mathematical models describing the pharmacometric properties of the artemisinin antimalarials have previously assumed a simple and direct relationship between drug exposure and malaria parasite killing.^3–6^ Because drugs in this class are all eliminated very rapidly, these models predicted that more sustained exposure (created by slowing drug clearance, administering artemisinins by constant infusion or by frequent dosing) would enhance parasite clearance, and thus improve therapeutic responses. More frequent dosing was proposed as a solution to the threat posed by artemisinin-resistant *P. falciparum* infections.^6^ However, clinical studies did not confirm these predictions.^9^ There was no significant advantage in terms of parasite clearance or cure rate from giving the artemisinin derivatives more than once daily despite their rapid elimination. The simple pharmacometric models were therefore inadequate.

As our earlier simple pharmacometric model^4^ did not predict the observed dose–response relationships (i.e. the failure of frequent dosing to improve therapeutic responses), we have modified its structure. The new proposed model incorporated the hypothesis that some young *P. falciparum* parasites are injured in the presence of artemisinins, stop growing, and become temporarily insensitive to the drug. A small proportion of these injured parasites can recover and return to their normal, drug-sensitive state. Whether this unresponsive (‘injury’) state causing a temporary arrest in development is the same or a similar process to the well-described ‘dormancy’ phenomenon is not specified. Both processes can explain recrudescence following standard treatments.^12–14^ The revised model was fitted to parasite count data obtained from patients in western Cambodia (where artemisinin resistance was prevalent) and western Thailand (before the main emergence of artemisinin resistance there). The model captured satisfactorily the dynamics of *P. falciparum* parasites in patients receiving artesunate monotherapy once daily for 7 days. As reported previously,^21^ the major difference between parasites from Cambodia, which were artemisinin resistant, and those from western Thailand, which were sensitive, was the more than 10-fold estimated reduction in ring stage killing. Trophozoite stage killing was estimated as similar but schizont killing was reduced by about 45%. This reduction in parasite killing at the schizont stage in artemisinin-resistant parasites is consistent with a recent *in vitro* study.^30^ Estimated recovery rates were slightly but not significantly different between the two sites. If the model is correct, this suggests that recovery rates from parasite injury are not affected substantially by the mechanisms involved in artemisinin resistance.

Artemisinin resistance in *P. falciparum* is characterized by reduced parasite clearance *in vivo*.^21^ This reflects reduced susceptibility of the circulating ring stage parasites.^31^ Artemisinin resistance in field isolates is causally associated with mutations in the propeller region of the *Pf*kelch gene located on chromosome 13 (K13).^32^ This causal association has been confirmed in transfection studies, although the contribution of other genes (often described collectively as the genetic background) is substantial.^33^ Artemisinin resistance is thought to involve altered parasite cellular responses rather than receptor or transporter alterations, but the exact mechanism is unclear. As the more mature stages of K13 mutant *P. falciparum* isolates remain sensitive to the artemisinin derivatives, the drugs are still efficacious in clinical practice but, as they kill fewer parasites per asexual cycle, the therapeutic responses are diminished.^34,35^ Longer exposures from longer courses over three or four asexual cycles improve therapeutic responses in artemisinin-resistant infections, but giving the drugs more frequently than once daily does not.^9,34^ The lower predicted death rates of the asexual ring stage parasites in the artemisinin-resistant infections is supported by extensive experimental investigations^13–19,31^ and the strong correlations between specific ring stage *in vitro* susceptibility evaluations, K13 mutations and slow parasite clearance.^34^ The postulated drug-unresponsive (‘injury’) state incorporated in this model persisted between 2 and 50 h. Nearly all damaged parasites were ultimately cleared. The estimated parasite recovery rate was about 1 in 740–850 unresponsive parasites per 24 h. This is comparable to proportions found in previous *in vitro* studies.^13,36^ The estimated recovery lag time was between 2 and 2.4 h, indicating that the parasites that recovered did not do so immediately, but after a delay of a few hours. Consequently, the duration of drug refractoriness was the sum of duration of the injured state and this lag time.

Dormancy induced by artemisinin antimalarials has been modelled previously.^13,14,24^ However, the definition or the interpretation of the dormancy-like state in these models varied. For example, Hoshen *et al.*^24^ modelled dormancy as parasites that were unobservable and insensitive to the drug but reappeared in the simulation at their pre-dormant stage after an interval. Gordi *et al.*^3^ proposed a model structure that included sensitive, insensitive and injured stages for parasites during treatment in measurable and unmeasurable compartments. In their model, parasites injured by the antimalarial drug are removed by the spleen. Jones *et al.*^7^ proposed a model structure in which dead parasites are not removed immediately, but accumulate in the circulation after exposure to the drug before being removed by the spleen. In our model, the term ‘injury state’ was chosen specifically to describe the process of cellular damage, developmental arrest and temporary refractoriness to further dosing. Further laboratory studies are needed to determine whether this is the same process as the previously described ‘dormancy’.

Our model was developed to account for the failure of frequent dosing regimens to accelerate parasite clearance or enhance cure rates following artemisinin-containing antimalarial drug treatments (as predicted by our earlier simple pharmacometric model).^4^ While the revised structure did this satisfactorily, there are several limitations to this modelling exercise. Many of the pharmacodynamic parameter values in this model have not been measured directly so the system is unidentifiable. It has also simplified greatly the complex relationship between parasite stage of development, and time and intensity of drug exposure, and it has assumed homogeneous parasite stage distributions and multiplication and elimination kinetics. It has also modelled only DHA plasma concentrations, thereby ignoring the small contribution of the plasma concentrations of unhydrolysed parent drug artesunate to parasiticidal effects. These are all oversimplifications. Although the model can reproduce the observed data in this pharmacometrics study, this does not mean that it has explained the underlying biology (i.e. the model may not be correct). Nevertheless, it does propose a relatively simple hypothesis that is consistent with observations, and is testable. But there may well be other hypotheses for which other models would fit equally well with the data. We propose that, as a minimum, such models should be capable of reproducing both delayed parasite clearance in artemisinin-resistant infections and the failure of frequent dosing to augment therapeutic responses.^8^

In conclusion, a new within-host pharmacometric model is proposed, which supports the hypothesis that parasites enter a temporary drug refractory or ‘injury’ state after contact with artemisinin antimalarials, which is followed either by parasite death or reactivation. The model fitted the observed sequential parasite density data from patients with artemisinin-resistant and -sensitive *P. falciparum* infections, and it confirmed the known dose–response relationship for reduced ring stage activity in artemisinin-resistant infections.

## Supplementary Material

dkad219_Supplementary_Data

## Supplementary data

Figures S1 to S4 and Tables S1 to S3 are available as Supplementary data at *JAC* Online.
