# Supplementary material for: An artesunate pharmacometric model to explain therapeutic responses in falciparum malaria
Source: J Antimicrob Chemother. 2023 Jul 20;78(9):2192–202. doi: 10.1093/jac/dkad219 (PMC10477127; doi:10.1093/jac/dkad219)

**Supplementary**

Table S1: The estimated pharmacokinetic parameters derived from the plasma DHA concentrations from each patient

| **PID** | **time at max Conc (**$\boldsymbol{t}_{\boldsymbol{m}}$**) (hours)** | **Max concentration(**$\boldsymbol{c}_{\boldsymbol{m}}$**)(ng/ml)** | **Elimination rate (**$\boldsymbol{k}$**) (/hour)** | **Site** |
| --- | --- | --- | --- | --- |
| M001 | 0.76 | 2233.65 | 1.05 | Wang Pha |
| M003 | 3.58 | 214.10 | 0.89 | Wang Pha |
| M005 | 0.75 | 1467.06 | 1.01 | Wang Pha |
| M006 | 1.40 | 1196.41 | 1.07 | Wang Pha |
| M007 | 0.74 | 857.15 | 0.78 | Wang Pha |
| M011 | 2.51 | 1133.77 | 1.47 | Wang Pha |
| M012 | 1.05 | 468.96 | 0.46 | Wang Pha |
| M013 | 0.45 | 532.87 | 0.65 | Wang Pha |
| M014 | 0.48 | 1360.17 | 1.20 | Wang Pha |
| M021 | 1.09 | 654.98 | 1.23 | Wang Pha |
| M023 | 1.16 | 960.78 | 0.88 | Wang Pha |
| M024 | 1.16 | 1326.49 | 1.37 | Wang Pha |
| M026 | 1.15 | 1086.55 | 1.04 | Wang Pha |
| M030 | 1.04 | 551.68 | 0.92 | Wang Pha |
| M032 | 1.10 | 1423.83 | 0.82 | Wang Pha |
| M035 | 0.55 | 1350.98 | 1.29 | Wang Pha |
| M036 | 0.89 | 278.56 | 0.33 | Wang Pha |
| M037 | 1.04 | 933.01 | 1.00 | Wang Pha |
| M039 | 2.03 | 461.77 | 0.97 | Wang Pha |
| P002 | 0.88 | 743.88 | 1.13 | Pailin |
| P003 | 0.49 | 1119.99 | 0.70 | Pailin |
| P006 | 4.26 | 379.47 | 1.16 | Pailin |
| P008 | 0.95 | 675.77 | 0.56 | Pailin |
| P009 | 1.93 | 1574.32 | 0.74 | Pailin |
| P011 | 1.10 | 752.57 | 0.88 | Pailin |
| P013 | 0.61 | 891.70 | 1.54 | Pailin |
| P014 | 1.06 | 986.04 | 0.60 | Pailin |
| P018 | 1.22 | 645.94 | 1.12 | Pailin |
| P020 | 1.27 | 1105.08 | 0.89 | Pailin |
| P021 | 2.17 | 901.20 | 1.27 | Pailin |
| P022 | 2.48 | 731.25 | 0.70 | Pailin |
| P023 | 0.52 | 962.26 | 1.14 | Pailin |
| P025 | 0.96 | 1146.51 | 0.97 | Pailin |
| P030 | 3.26 | 662.98 | 1.91 | Pailin |
| P031 | 3.17 | 398.19 | 0.88 | Pailin |
| P033 | 1.26 | 139.97 | 0.31 | Pailin |
| P035 | 1.08 | 1019.50 | 1.33 | Pailin |
| P037 | 3.01 | 255.69 | 0.61 | Pailin |
| P039 | 0.41 | 2983.38 | 0.88 | Pailin |

Table S2: Mean (SD) of the pharmacokinetic parameters derived from the plasma DHA concentrations

| **Site** | $\boldsymbol{t}_{\boldsymbol{m}}$ | $\boldsymbol{c}_{\boldsymbol{m}}$ | $\boldsymbol{k}$ |
| --- | --- | --- | --- |
| Wang Pha | 1.21(0.76) | 973.30(505.75) | 0.97(0.29) |
| Pailin | 1.61(1.10) | 903.78(595.28) | 0.97(0.37) |

Table S3: Median (25^th^ ,75^th^ percentiles) of the simulated parasite clearance times as shown in Figure 6

| **Site** | **Every 24 hours** | **Every 12 hours** |
| --- | --- | --- |
| **Wang Pha** | 30.0 (20.75, 50.75) | 23.0 (16.25, 44.25) |
| **Pailin** | 73.5 (53.5, 93.5) | 70.5 (51.5, 89.5) |

Figure S1: The results from fitting the proposed model to the parasite clearance data from patients who received artesunate monotherapy in Wang Pha, Thailand. In each plot, the blue dots represent the observed data and the grey line represents the median of the model outputs. The light-grey shaded area represents the 95% credible intervals (CIs).
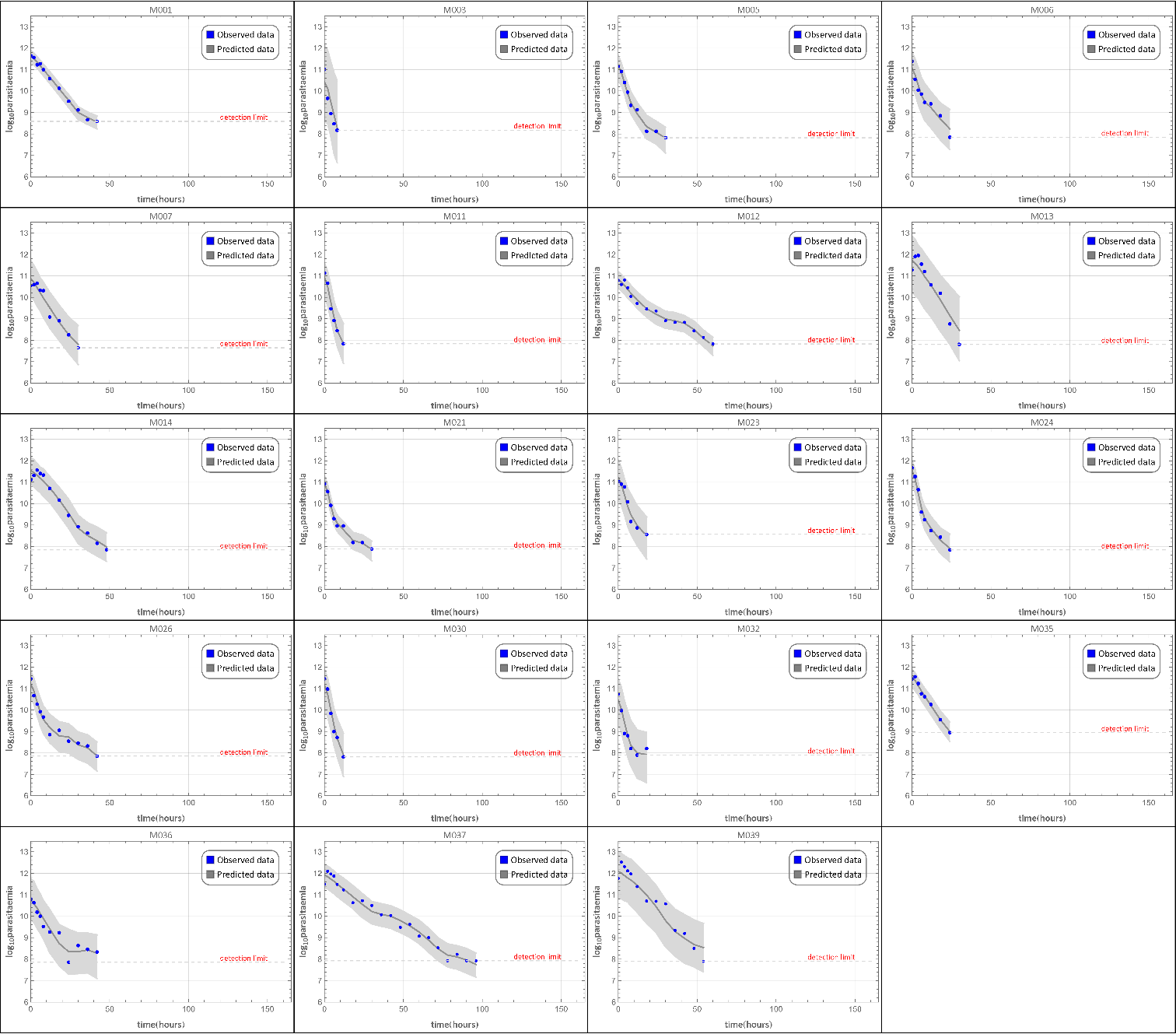


Figure S2: The results from fitting the proposed model to the parasite clearance data from patients who received artesunate monotherapy in Pailin, Cambodia. In each plot, the blue dots represent the observed data and the grey line represents the median of the model outputs. The light-grey shaded area represents the 95% credible intervals (CIs).


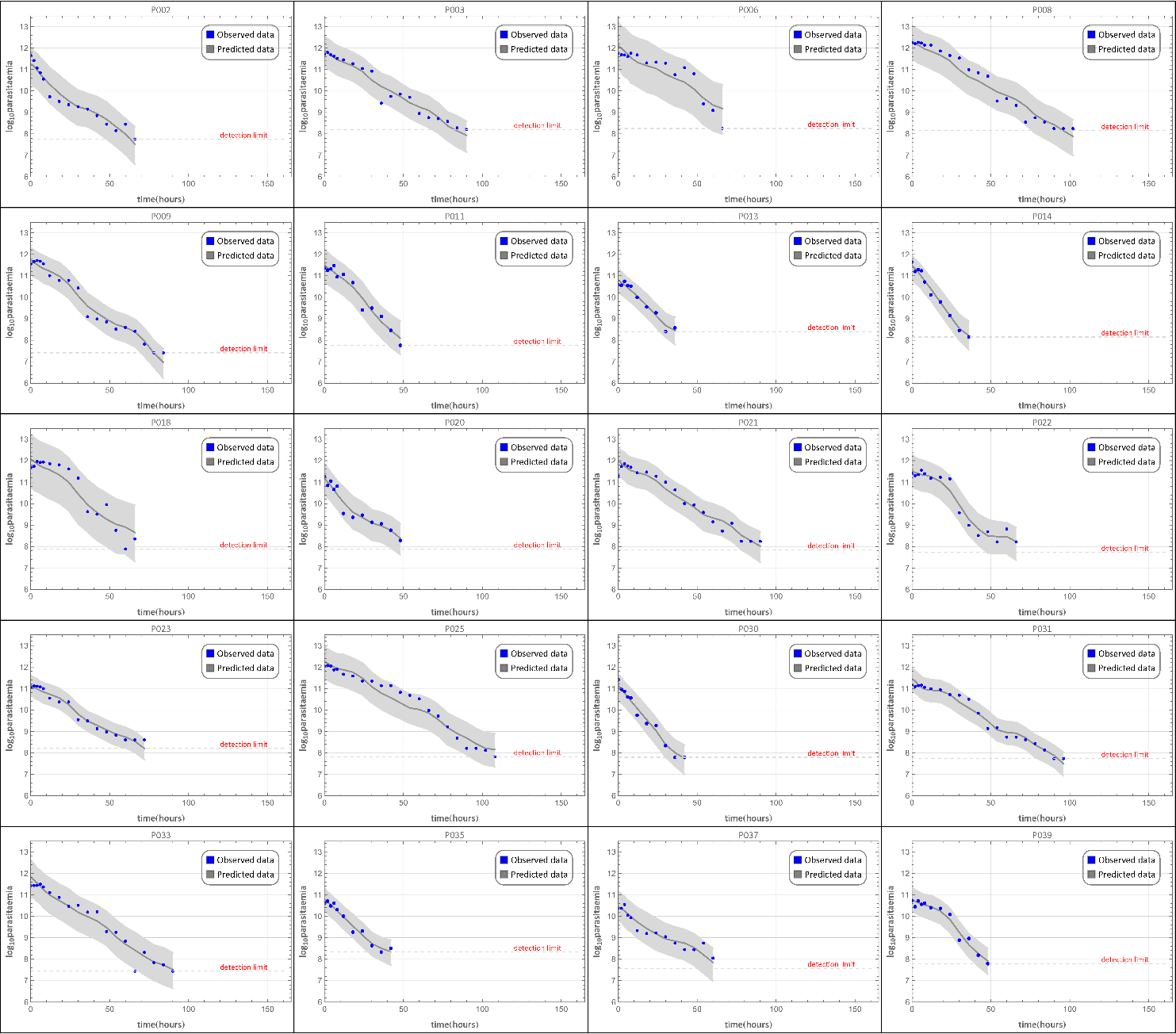


Figure S3: The results from the dose-splitting simulation in Wang Pha, Thailand. The black dots represent the observed data, the purple lines represent the median of the model outputs for taking the drug every 24 hours and the green lines represent the model outputs for taking the drug every 12 hours.


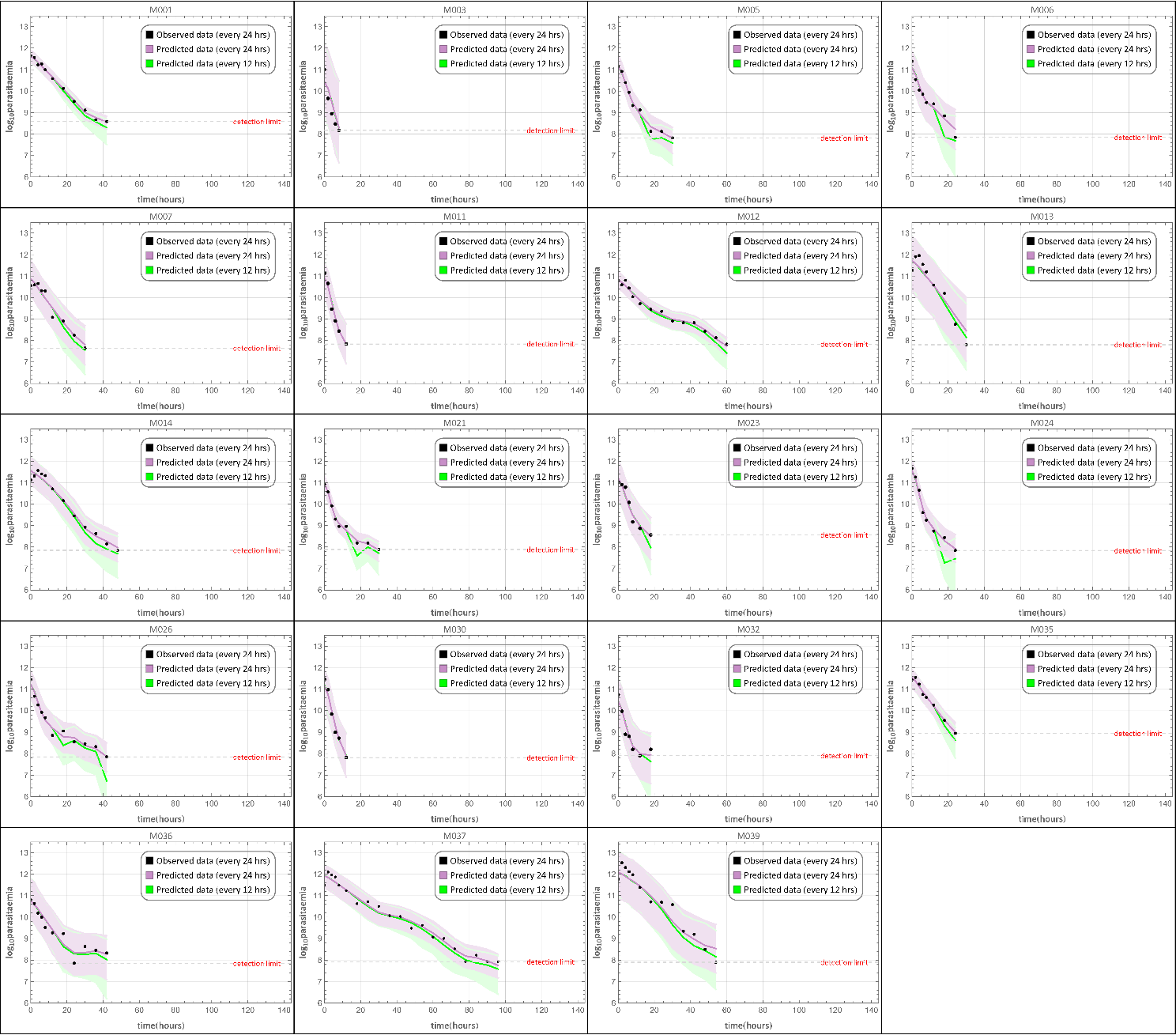


Figure S4 The results from the dose-splitting simulation in Pailin, Cambodia. The black dots represent the observed data, the purple lines represent the median of the model outputs for taking the drug every 24 hours and the green lines represent the model outputs for taking the drug every 12 hours.


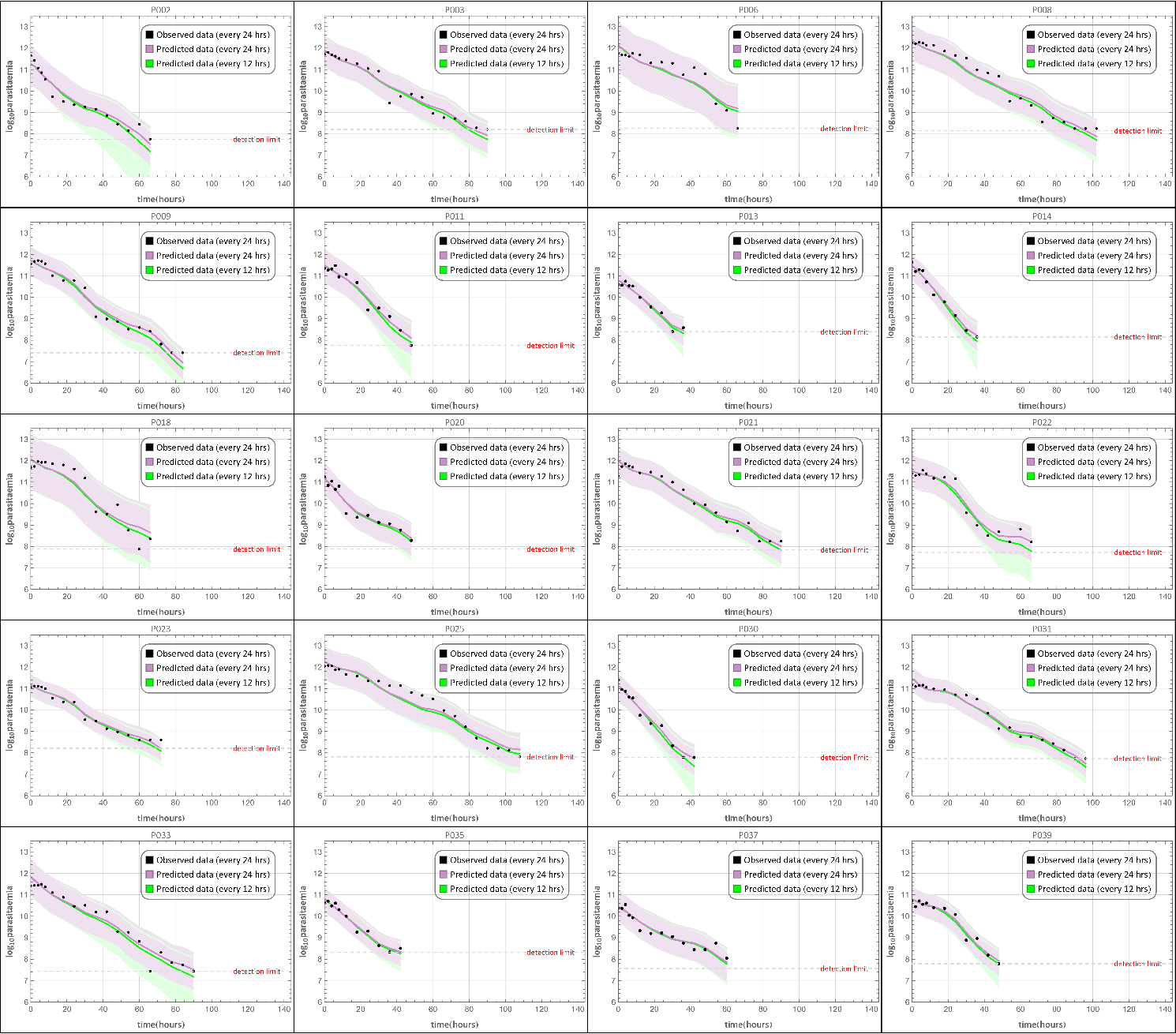

Supplement: dkad219_Supplementary_Data [file dkad219_supplementary_data.zip › Supplementary Saralamba et al.docx]
