# Supplementary figures and images for: An artesunate pharmacometric model to explain therapeutic responses in falciparum malaria
Source: J Antimicrob Chemother. 2023 Jul 20;78(9):2192–202. doi: 10.1093/jac/dkad219 (PMC10477127; doi:10.1093/jac/dkad219)

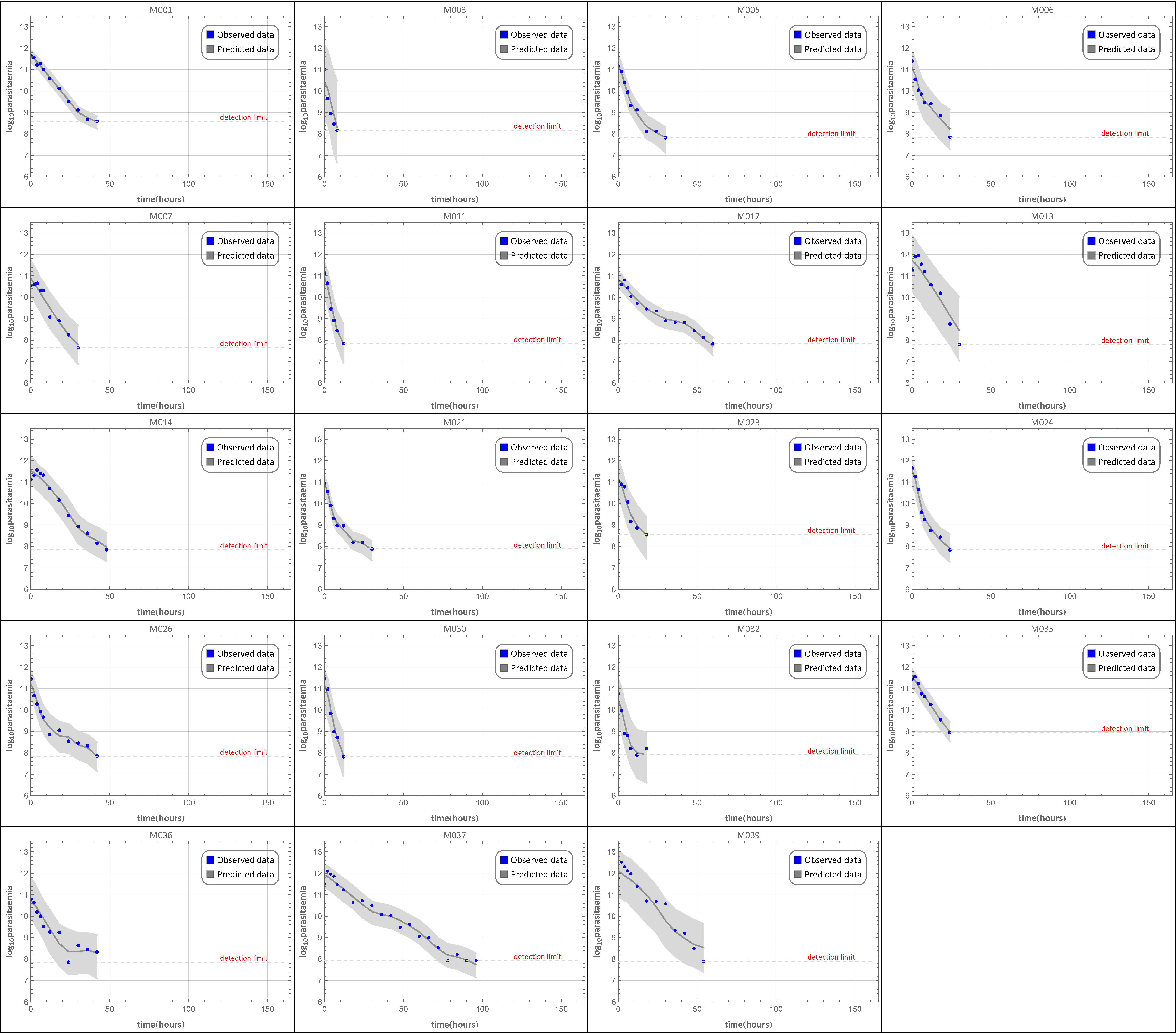

Supplement: dkad219_Supplementary_Data [file dkad219_supplementary_data.zip › Figure_S1.png]

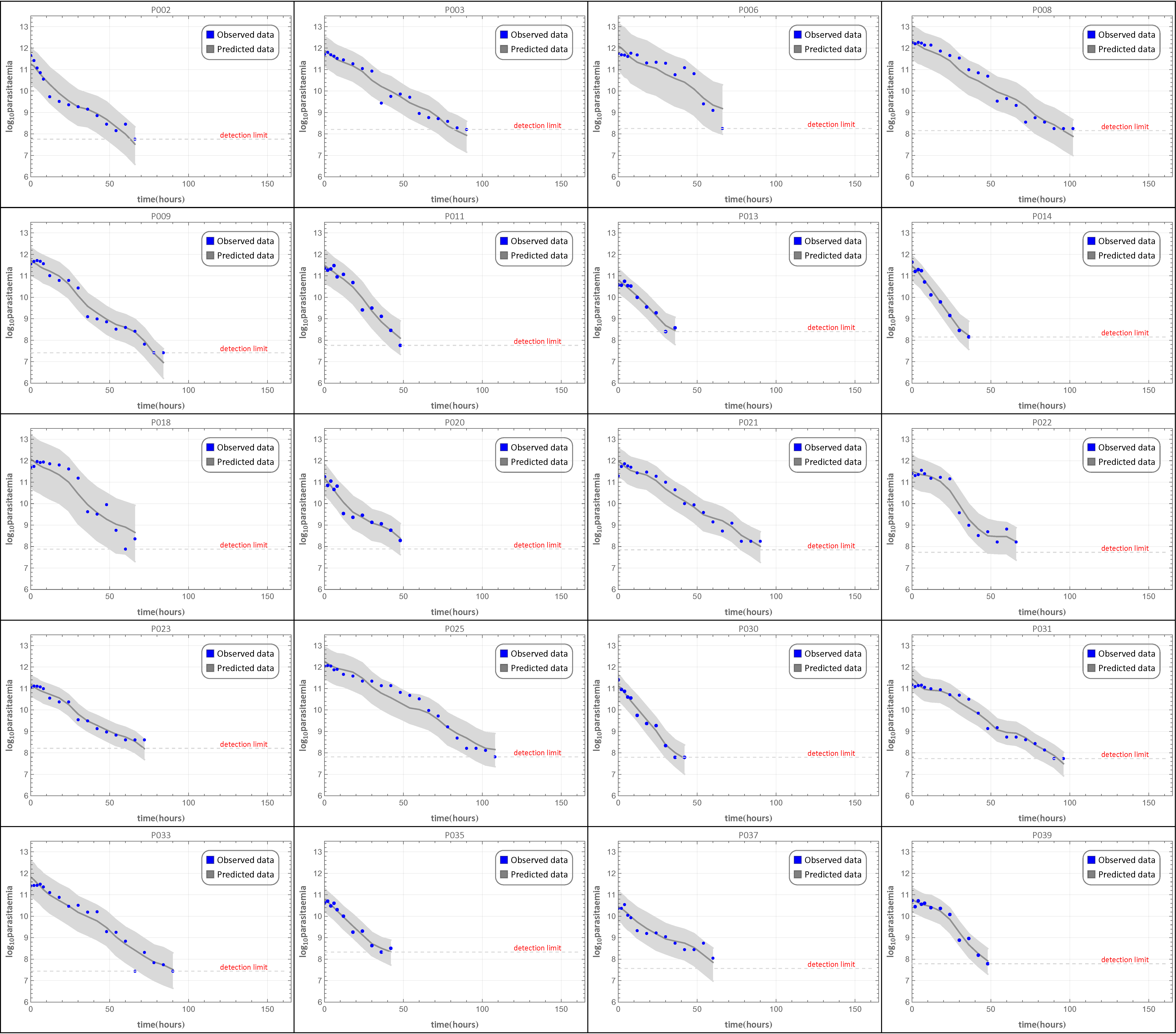

Supplement: dkad219_Supplementary_Data [file dkad219_supplementary_data.zip › Figure_S2.png]

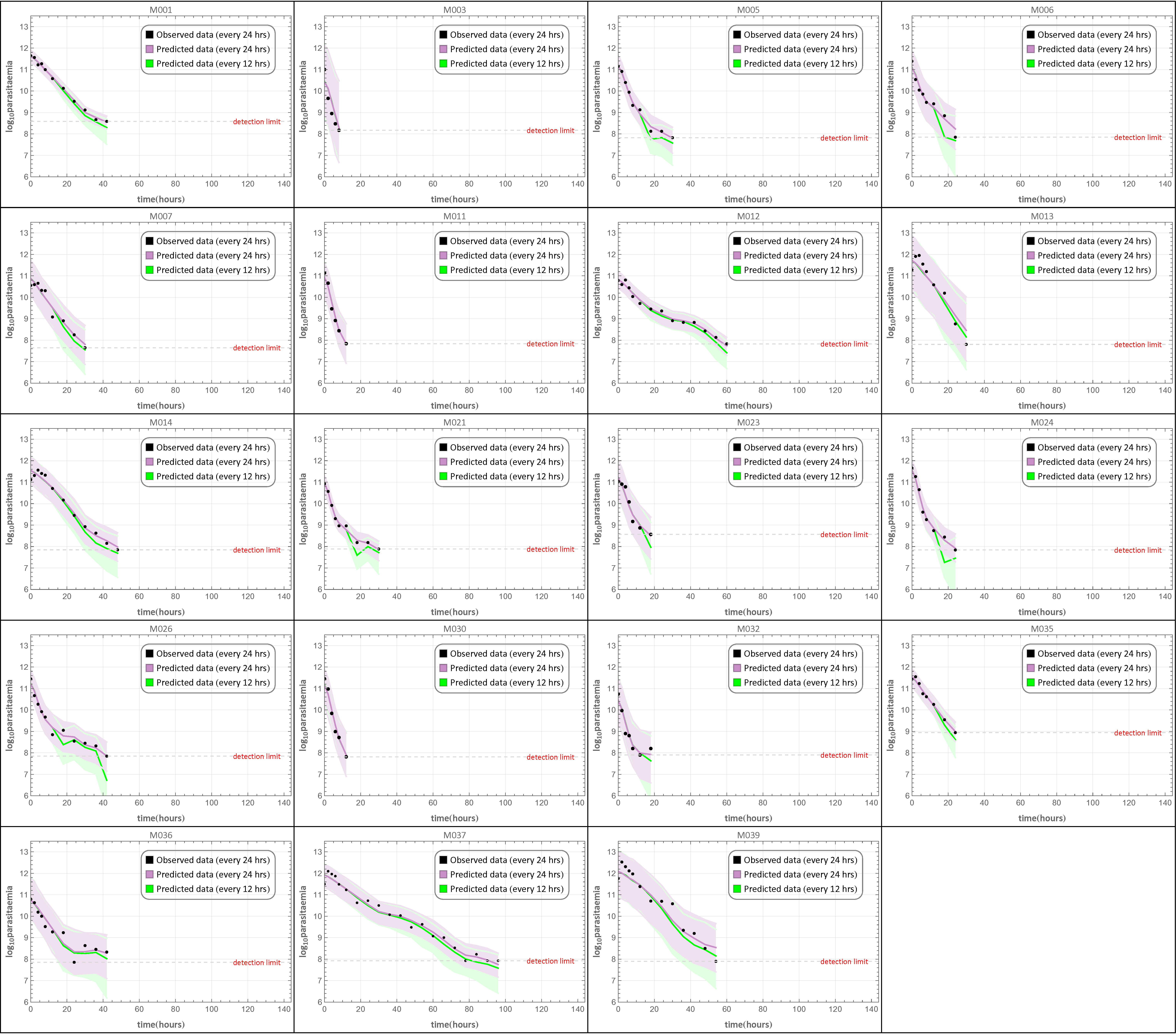

Supplement: dkad219_Supplementary_Data [file dkad219_supplementary_data.zip › Figure_S3.png]

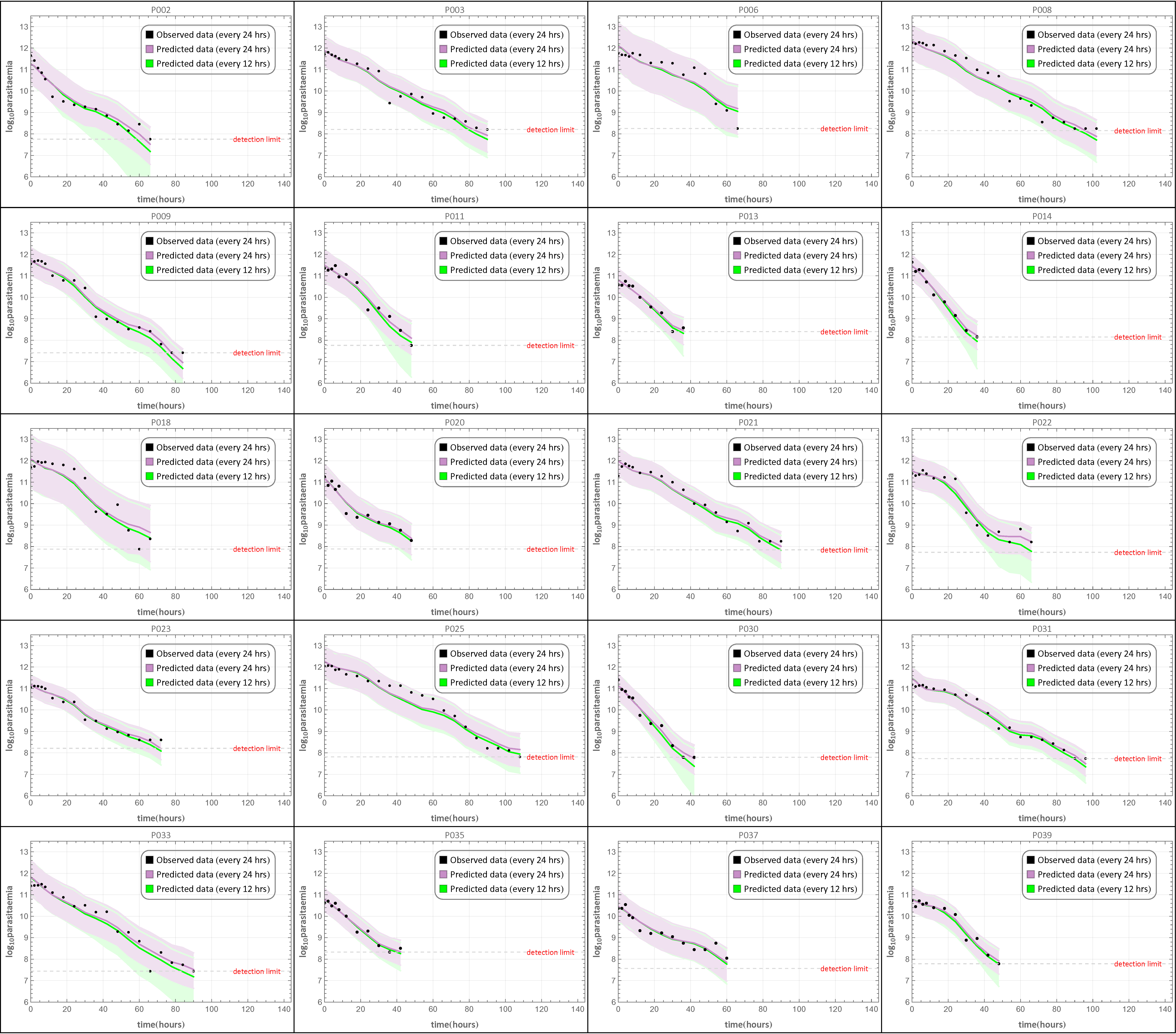

Supplement: dkad219_Supplementary_Data [file dkad219_supplementary_data.zip › Figure_S4.png]
